# Supplementary material for: Genome-Wide Characterization and Expression Analysis of KH Family Genes Response to ABA and SA in Arabidopsis thaliana
Source: Int J Mol Sci. 2022 Jan 3;23(1):511. doi: 10.3390/ijms23010511 (PMC8745409; doi:10.3390/ijms23010511)
Supplement: Supplementary file 1 [file ijms-23-00511-s001.zip › Figure S3.pdf]

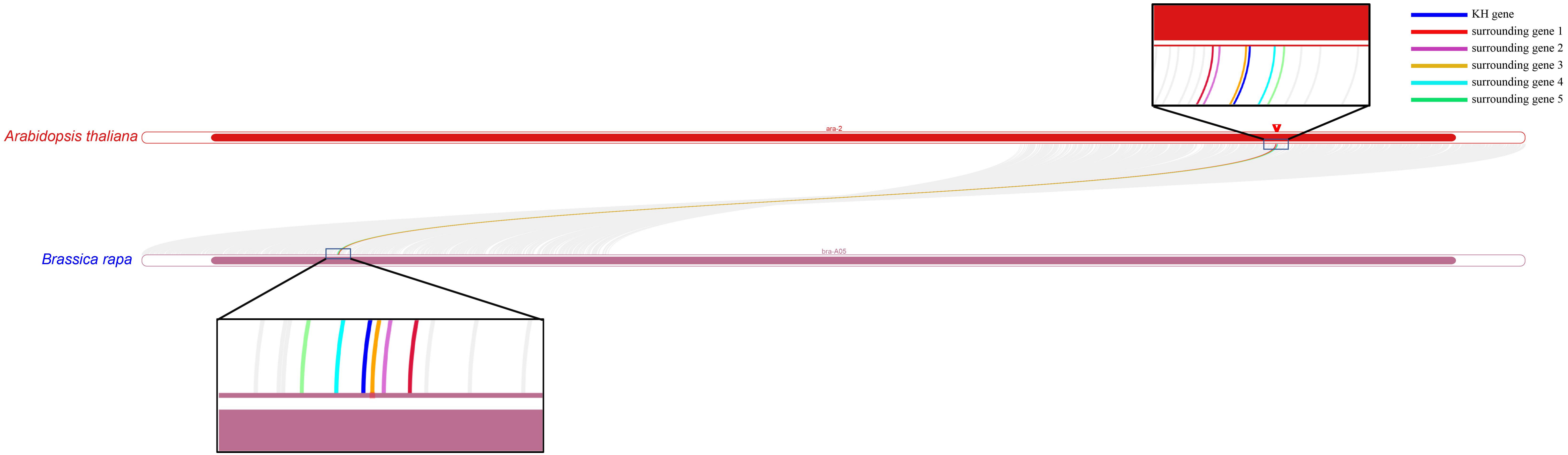

Figure S3: An example of *AtKH9-BrKH1* to display synteny blocks, the blue line refers to *AtKH9-BrKH1*, and the other colored lines refer to the surrounding genes of *AtKH9* and *BrKH1*.
