# Supplementary material for: Genome-Wide Characterization and Expression Analysis of KH Family Genes Response to ABA and SA in Arabidopsis thaliana
Source: Int J Mol Sci. 2022 Jan 3;23(1):511. doi: 10.3390/ijms23010511 (PMC8745409; doi:10.3390/ijms23010511)
Supplement: Supplementary file 1 [file ijms-23-00511-s001.zip › Figure S4.pdf]

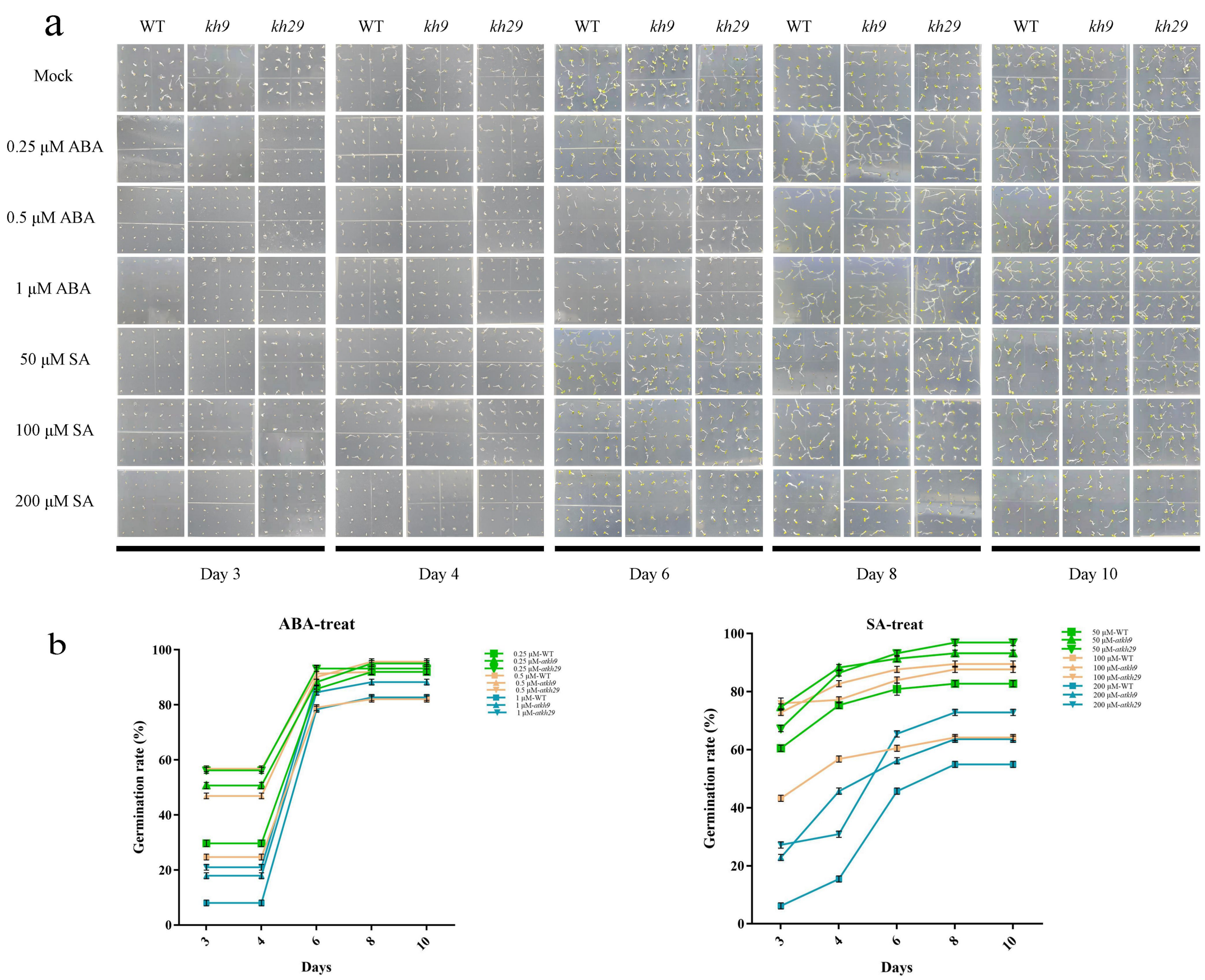

Figure S4: The seed germination rates of *atkh9* and *atkh29* under ABA and SA treatments. (**a**). Picture of 3 d, 4 d, 6 d, 8 d, 10 d seedlings of wild-type, *atkh9* and *atkh29* on the medium containing 0.25 μM, 0.5 μM, 1 μM ABA, and 50 μM, 100 μM, 200 μM SA. (**b**) Statistics of the germination rates of wild-type, *atkh9* and *atkh29*. Error bars indicated SD.
