# Supplementary figures and images for: Genome-Wide Characterization and Expression Analysis of KH Family Genes Response to ABA and SA in Arabidopsis thaliana
Source: Int J Mol Sci. 2022 Jan 3;23(1):511. doi: 10.3390/ijms23010511 (PMC8745409; doi:10.3390/ijms23010511)

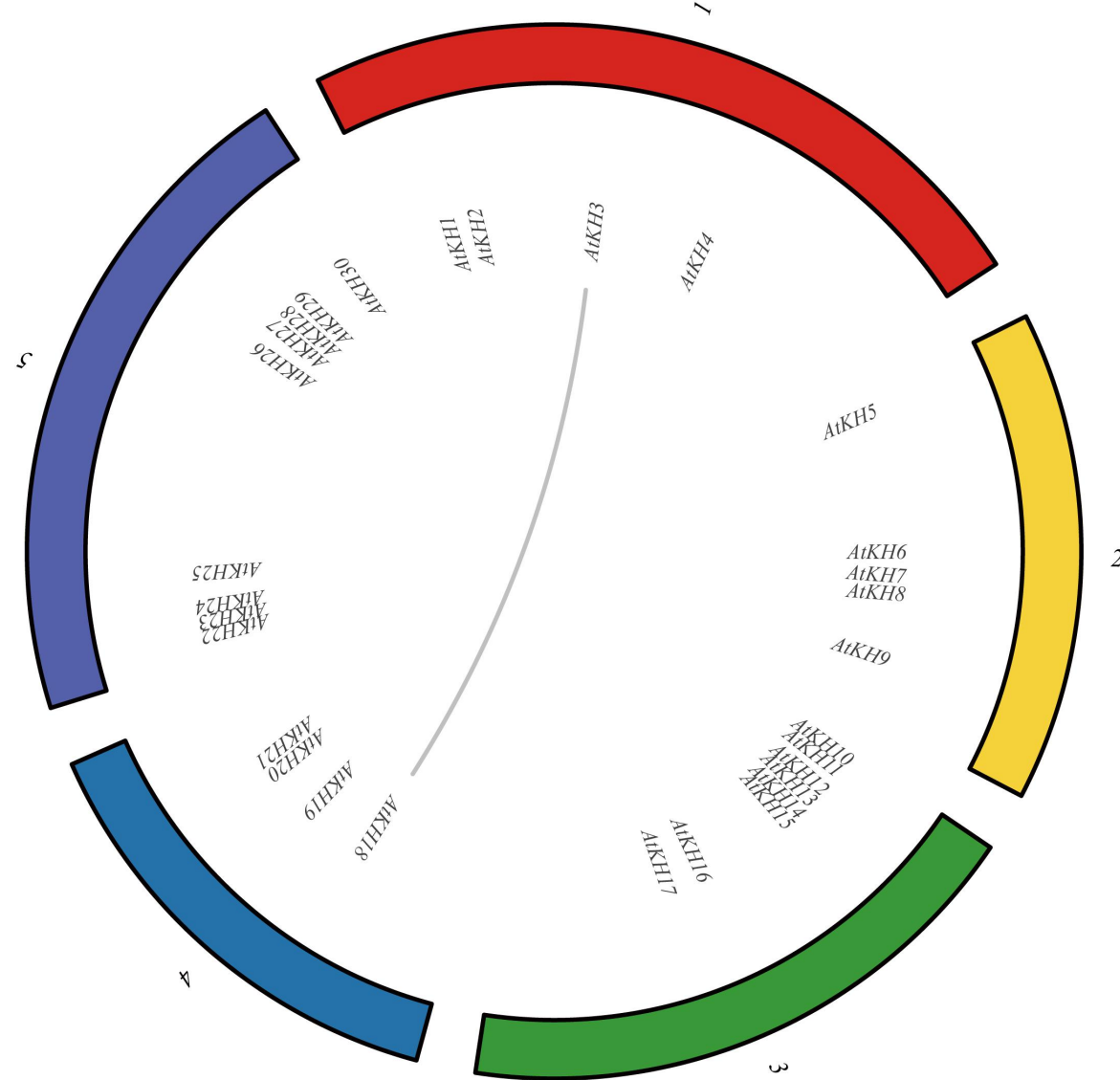

Figure S1: The distribution of 30 *Arabidopsis thaliana* KH domain genes on the chromosome.

Supplement: Supplementary file 1 [file ijms-23-00511-s001.zip › Figure S1.pdf]

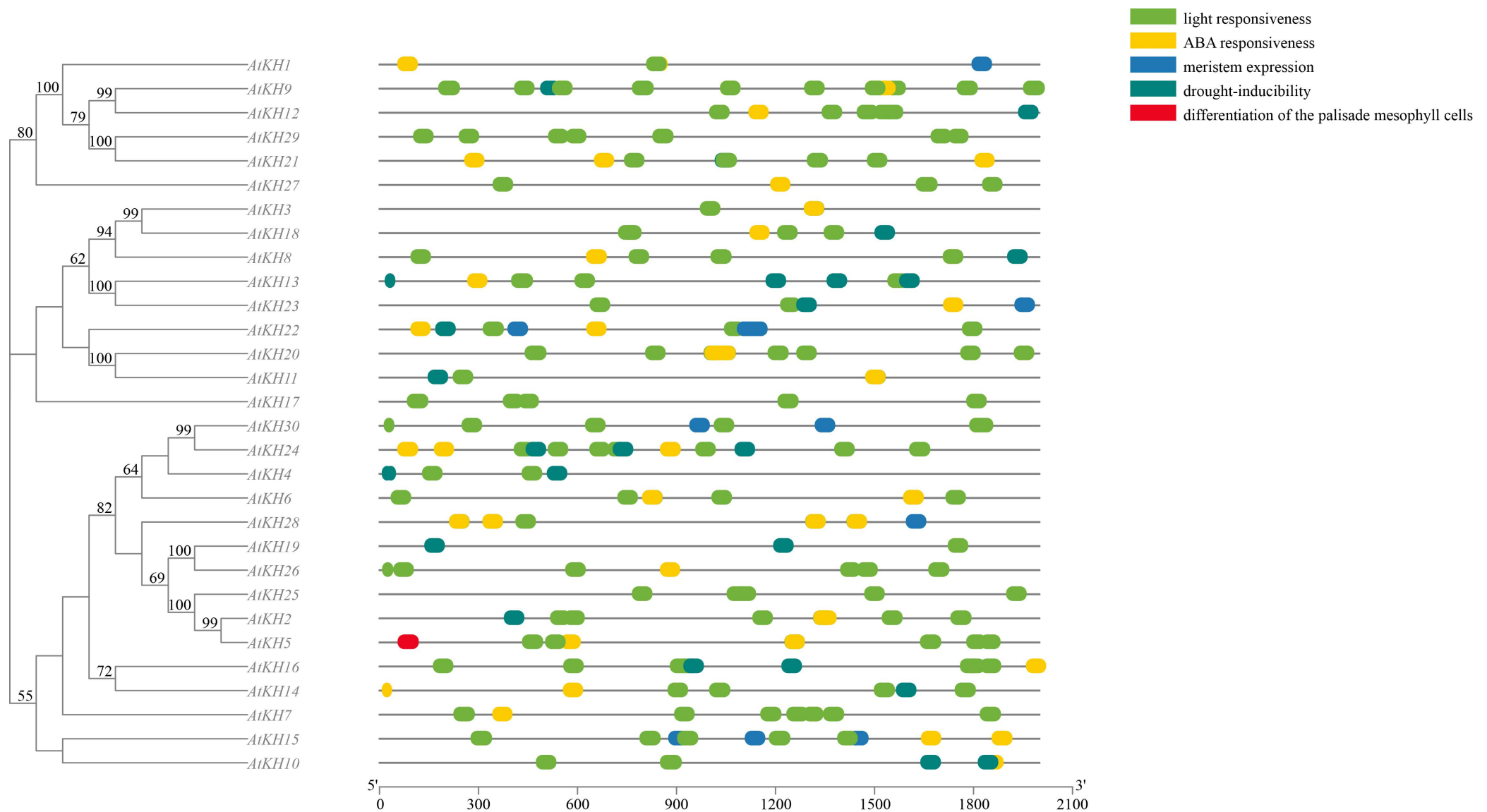

Figure S2: The *cis*-elements in the promoter region of KH family in *Arabidopsis thaliana*.

Supplement: Supplementary file 1 [file ijms-23-00511-s001.zip › Figure S2.pdf]
